# Anæsthesia in Dental Surgery—Its History and Introduction into Europe

**Published:** 1855-04

**Authors:** J. Robinson


### ARTICLE II.

*Anæsthesia in Dental Surgery—Its Historg and Introduction into Europe.* By J. Robinson, D. D. S., &c.

Sulphuric ether had been employed by the medical profession as a stimulant and antispasmodic for many years, but inhalation of its vapor was first proposed by Dr. Pearson in 1795. In 1796, in a contribution to the work of Dr. Beddoes on "Factitious Airs," a communication will be found from a patient of Dr. Thorton's, suffering at the time from attacks of catarrh, who says, "It gave almost immediate relief, both to the oppression and pain in the chest, and upon a second trial, in which two spoonfuls were inhaled, immediate relief followed and I very soon fell asleep." Sir Benjamin Brodie performed some experiments on Guinea pigs and other animals, with vapor of sulphuric either, which were narcotized and killed, and from this circumstance he doubted its safety. M. Ducos also furnishes an account of a number of experiments upon animals, all of which presented similar phenomena to those which have since been observed in the human subject.

The attention of scientific men was indeed directed more to the inhalation of the vapor of ether as a remedial agent, than to the production of insensibility, under which severe surgical operations might be performed without pain. In the year 1846,

Dr. Morton, a dentist, of Boston, in the United States of America, commenced a series of experiments upon animals with the vapor of ether, mixed with atmospheric air, and on September 30th, he induced a person desirous of having a tooth extracted, to inhale the vapor for the purpose of avoiding pain, the result of which was perfectly satisfactory. In November, after repeated trials, he obtained the sanction of the medical officers of the Massachusetts General Hospital to administer this agent in some severe surgical operations, having previously confined his experiments to his own special practice as a dentist. The exhibition of the vapor at the public hospital was attended with uniform success. Fearful surgical operations were performed, the unconscious patients awoke as from a pleasant dream, the recovery was rapid and unattended with any more serious results than the excitement and subsequent debility produced by a powerful stimulant. A few weeks only elapsed before anæsthesia by etherization received the full and unqualified recognition from the heads of the medical staff of the hospital. It is, therefore, to Dr. Morton, a dentist, that humanity is indebted for the practical application of ether to the production of anæsthesia at will during surgical operations. While these investigations were proceeding at the Massachusetts hospital at Boston, my esteemed friend Dr. F. Booth received a private communication from Dr. Bigelow, dated Boston, November 28th, 1846, in which he informed him of "a new anodyne process lately introduced here, which promises to be one of the most important discoveries of the present age. * * * * Consisting of the inhalation of the vapor of ether to the point of intoxication, rendering the patient insensible to pain during surgical operations, and other causes of suffering."

The above extract from the private letter to Dr. Booth with a newspaper report, constituted the whole of the intelligence on the effects of ether received in England on the 17th Dec., 1846. Dr. Booth communicated this intelligence to myself and others on the 19th, at his house, and in the presence of his family, I etherized a young lady, and extracted a molar tooth; this was the first operation, under the influence of ether, performed in

England, which was subsequently reported to the medical journals; in a few days I introduced the new agent at several of the metropolitan hospitals, and employed it for dental operations in my own practice. Fortified with increased experience, and the opinions of many of our eminent medical men, I had no hesitation in administering the vapor in long and protracted surgical operations after, by increased facilities and careful observations, I had noted the various stages of etherization as an index when the operation should be commenced. I observed after the excitement had passed and semi-unconsciousness commenced that the eyes were generally open, the pupils dilated, but as perfect narcotism approached, the pupil became contracted, and was fixed elevated beneath the upper eye-lid, the patient was kept in this condition during the time occupied in the performance of an operation, by the occasional administration of the vapor whenever the pupil dilated and began to recover its sensibility to light, and by carefully observing this condition of the eye, I was enabled to retain the patient fully under the influence of the vapor so long as was necessary.

In a few months the great majority of our operating surgeons recognized the principle that sensibility might be suspended in most individuals for a time, without subsequent injury to their general health, and etherization was commonly employed at most of the public charities and in private practice. By many operators, however, the employment of ether was considered objectionable, as its administration was frequently accompanied by prolonged excitement, and difficulty in managing the patient. It also occupied, in many cases, considerable time before the patient could be brought fully under its influence. After producing irritation and coughing, and to some patients the smell and taste were objectionable, the breath retained the odor for many hours. These were considered objections to its employment in the minor cases of surgery, and many other substances were experimented upon in the hope of discovering a more suitable agent; without any satisfactory results, until Dr. Simpson, of Edinburg, showed that chloroform would produce similar effects, and was considered by him equally safe, and not open to those objections previously urged against ether.*

Dr. Simpson, in a pamphlet written at the time of its introduction, when speaking comparatively of ether and chloroform as anæsthetic agents, says,

"1st. A greatly less quantity of chloroform, than of ether, is requisite to produce the anæsthetic effect: usually from a hundred to a hundred and twenty drops of chloroform only being sufficient, and with some patients much less. I have seen a strong person rendered completely insensible by six or seven inspirations of thirty drops of the liquid.

2d. Its action is much more rapid and complete, and generally more persistent. I have almost always seen from ten to twenty full inspirations suffice. Hence the time of the surgeon is saved; and that preliminary stage of excitement which pertains to all narcotizing agents, being curtailed, or indeed practically abolished, the patient has not the same degree of tendency to exhilaration and talking.

3d. Most of those who know from previous experience the sensations produced by ether inhalation, and who have subsequently breathed the chloroform, have strongly declared the

* Although the results attending the introduction of anæsthetic agents have met with universal approbation of the most scientific and enlightened medical and surgical practitioners in Europe, and is now universally employed in our hospitals, the director general of the medical staff, now in the Crimea, has thought proper, in opposition to the most learned and experienced operators in Europe, to recommend its not being employed in surgical operations on our brave and unfortunate soldiers now battling in the Crimea. It is to be hoped that some humane and spirited member of the legislatue will bring the subject before parliament, by which means the public and the profession will ascertain upon whose authority (besides his own) Dr. Hall has excluded the use of chloroform from the poor soldier's pharmacopœia. The following is part of this wise man's address to the medical staff: "Dr. Hall takes this opportunity of cautioning medical officers against the use of chloroform in the severe shock of serious gunshot wounds, as he thinks few will survive where it is used. But as public opinion, founded perhaps on mistaken philanthropy, he knows, is against him, he can only caution medical officers, and entreat they will narrowly watch its effects, for, however barbarous it may appear, the smart of the knife is a powerful stimulant, and it is much better to hear a man bawl lustily than to see him sink silently into the grave."

inhalation and influence of chloroform to be far more agreeable and pleasant than those of ether.

4th. I believe that, considering the small quantity required as compared with ether, the use of chloroform will be less expensive than that of ether, more especially as there is every prospect that the means of forming it may be simplified and cheapened.

5th. Its perfume is not unpleasant, but the reverse, and the odor of it does not remain, for any length of time, obstinately attached to the clothes of the attendant, or exhaling in a disagreeable form from the lungs of the patient, as so generally happens with sulphuric ether.

6th. Being required in much less quantiity, it is much more portable and transmissible than sulphuric ether.

7th. No special kind of inhaler or instrument is necessary for its exhibition, a little of the liquid diffused upon the interior of a hollow shaped sponge or a pocket handerchief, or a piece of linen or paper, and held over the mouth and nostrils, so as to be fully inhaled, generally suffices in about a minute or two to produce the desired effect."

The substitute of chloroform for ether emanating from so high an authority as Professor Simpson, caused nearly as much curiosity in the medical world as the introduction of ether itself, the operating theatres of our hospitals were again crowded to witness the effects of the new agent, and the results proving satisfactory to the profession, anæsthesia by the inhalation of the vapor of chloroform was generally adopted at all the hospitals.

From my experience and judging from analogy, the effects of chloroform and ether in producing insensibility are nearly the same. We observe at the commencement of the inhalation of chloroform a degree of excitement which is curtailed by its rapid action as the patient approaches insensibility.

The administering the vapor occupies a much less time than that of ether, and may be said to be generally attended with more agreeable and pleasant sensations. Its effects, moreover, are more persistent than those of ether, and the patient is more quickly brought under the control of the operator. During the first inhalation, the pulse increases in frequency, but returns to its normal condition, when the patient is fully under its influence, when we have the same fixed state of the iris, and the same relaxation of the muscular fibre as with ether, and if the inhalation be prolonged beyond this point, stertorous breathing, profuse perspiration, pallid face and lips, accompanied with intense depression of the nervous system, while, in other patients, especially in females, severe hysterical attacks have followed its exhibition.

Dr. Snow, who has contributed many valuable papers upon the exhibition of ether and chloroform, and their influences upon the nervous system, after a series of experiments upon animals, divides anæsthesia into five degrees. "I divide etherization into five degrees, which may be called degrees of narcotism. The division was made according to symptoms which may be observed before an operation begins, leaving out of the classification the immunity from pain, which can only be ascertained during the operation, and which, curiously, does not correspond uniformly with the state of the patient in other respects. In that which I called the first degree, there is exhilaration, or altered emotions and sensations of some kind, but the patient still retains consciousness and volition. In the second degree, the mental functions may still be performed, but only in an irregular manner; there may be ideas of a dreaming kind, and voluntary efforts in accordance with them, or the patient may be passionate. When mental excitement occurs, it is chiefly in this degree in which the functions of the cerebral hemispheres seem to be impaired, but not yet abolished. In the third degree, these functions appear to be totally suspended, but those of the spinal cord, and its nerves still continue, to some extent; the orbiculars palpebrarum may control when the eye lids are touched; there may be other involuntary motions, resulting from external impressions, and groans or cries may occur, but no sounds of an articulate kind. There are also sometimes, in this degree, involuntary muscular contractions, as an effect of the vapor, apparently a kind of excitement of the spinal cord. In the fourth degree, no movement is obvious, except that of respiration which is unaffected by external impressions, and goes on regularly, though often with snoring, or with some degree of stupor. It would seem that the whole of the nervous centres are paralyzed by the vapor, except the medulla oblongata. In killing animals with vapors, I have observed the breathing to be difficult, or feeble, or otherwise impaired, before it finally ceased; this stage I call the fifth degree. There can be no doubt that these degrees of narcotism correspond with different proportions of vapor which are dissolved in the blood at the time. A certain quantity of vapor disturbs the functions of the cerebral hemispheres: an additional quantity appears altogether to suspend these functions, and to impair those of the spinal cord, and probably of the cerebellum, a still larger quantity to suspend the latter functions, but to leave the medulla oblongata more or less unaffected. As the vapor escapes from the blood by the lungs, its effects go off, the patient passes from the fourth degree to the third, from that to the second, and so on if the inhalation be not renewed."

*The Apparatus used for Administering Anæsthetic Agents.* There has been and still continues to be great diversity of opinion as to the best method of administering anæsthetic agents, some prefer the application of ether or chloroform by means of a cup of sponge, saturated with the fluid, and covered with a handkerchief held before the patient's mouth; a piece of sponge or folds of cotton wadding, containing the agent, is placed within the folds of a handkerchief and then held before the face, this we are aware is the method of many who employ these agents, and accords with the directions of Dr. Simpson, but in a somewhat extensive practice I have tried both methods, and prefer using a simple and inexpensive contrivance, furnished with an expirative valve, so as to prevent the expired air from being again inhaled; moreover, I have found that the agent can be administered more effectually and with a greater amount of certainty; the operator can more easily regulate the admission of atmospheric air, and the patient during the time is more under his control. The following is the apparatus we have employed for ether:

A vessel containing ether, with openings for the admission of atmospheric air. Wire tube, covered with silk, one inch in diameter, length with mountings 24 inches at the end. A piece of vulcanized India rubber is suspended the size of the tube· The chamber, or mask, which encloses the nose and mouth, this is screwed on the end of the tube, at the upper part of which is the expiratory valve, made of vulcanized India rubber; as the patient inhales, the valve in the tube opens, and admits the vapor, but as he exhales, it closes and opens the expiratory valve. The edge of the chamber, that part which comes in contact with the face, is flexible and easily adapted to the contour of the mouth and nose.

For administering chloroform, we have only to substitute the chamber and suspend a piece of sponge, on a hook in the interior, the chloroform being dropped upon the sponge.

*Directions for Inhalation.*—Ether or chloroform, as a *rule, should never* be administered immediately after a meal, or when any fluid stimuli have recently been taken into the stomach. The practitioner should, if possible, give directions or ascertain this fact from his patient before he proceeds to administer the vapor, for if neglected he will often find when the patient is narcotised, nausea, in most cases occurs, followed by vomiting, and the patient will infallibly suffer afterwards from debility and head-ache, and, therefore, it is more judicious to defer the period of an operation for some hours until the stomach is empty, and it is, moreover, necessary that the previous use of opium for any length of time should be also avoided.

I have invariably adopted the rule of permitting the patient to inhale the vapor gradually, diluted with a large proportion of atmospheric air, by keeping the apparatus at some distance from the face, at the commencement of the inhalation, so that he is thus brought unconsciously under its influence. As the inhalation proceeds, the flexible edges of the chamber should be closed around the mouth, and after a few seconds only, the pupil will be observed to become stationary, turn upwards towards the eyelids, and the patient be rendered perfectly insensible. By continuing the inhalation, the pupils will remain fixed, accompanied with perfect loss of vision and muscular power in the eyelids and of complete relaxation of the muscular system. At this point the inhalation should be discontinued, and the patient permitted to inhale atmospheric air, the operation may now be commenced, but if likely to be protracted, from unforeseen difficulties, the operator should observe carefully the pupil, and if this again begins to move quickly, he should apply the vapor for a few seconds, until the patient returns to the former condition, and this should be repeated from time to time, permitting the free inhalation of the atmosphere during the interval until the operation is finished. By carefully observing this condition of the eye and the pulse, I have kept patients fully under the influence of ether and chloroform for upwards of an hour in surgical operations, and in one instance nearly three hours, in instrumental delivery, without any injurious consequences. During the operation (especially if a protracted one) the head and face should be sponged with cold water. This should *always* be done afterwards, and if the reaction be not sufficiently rapid, galvanism should be applied to the extremities, and some diffusible stimuli be administered. I have also employed the inhalation of oxygen gas with excellent effect in cases when considerable debility has appeared after the exhibition of anæsthetic agents, but as this is seldom at hand, I have always had recourse to the galvanic battery, which every operator should have ready for use in his surgery. The application of one pole opposite the region of the heart, and the other to the corresponding part of the back or to the extremities, so as to send a current through this region, will be found a powerful stimulant, often more serviceable than diffusible stimulants, even presuming the patient possess sufficient power to swallow them. If during the inhalation the patient should show symptoms of asphyxia, stertorous breathing, or any other unfavorable symptom arising from the vapor, or from a want of atmospheric air, the inhalation should be immediately discontinued, cold water dashed on the face, artificial respiration employed if necessary, and when all unpleasant symptoms have disappeared the vapor can be again applied. The quantity of ether or chloroform necessary to produce the proper state of insensibility for an operation varies in different patients, some requiring not more than an ounce of the former, or twenty drops of the latter, whilst others will inhale as much as four ounces of ether or two or three drachms of chloroform. This in part depends upon the time occupied in producing insensibility. Considerable evaporation may occur before the inhaler is brought in contact with the mouth, but also on peculiarity of constitution. The average quantity employed, however, is two ounces of ether or a drachm and a half of chloroform. In the preceding observations we have been speaking of the inhalation of anæsthetic agents in severe and protracted surgical operations, in which it is necessary to continue the complete narcotization until the end of the operation. For dental purposes, for the extraction of one or two teeth, for example, it is rarely necessary to continue the exhibition of the vapor for any length of time, and only so far as to produce relaxation of the muscles of the jaw, except in those cases when a number of teeth and stumps are to be extracted, or some difficulty arises from some mal-formation in the fangs of a tooth, but in every case the operator must be guided by his own experience; he must narrowly observe the effect of the various degrees of narcotism, and seize the moment for his operation, when relaxation of the muscles of the jaw commences.* If the teeth or stumps are situated in the lower jaw, and especially when at the posterior part, the operator will frequently find that the hemorrhage obstructs his view of the remaining stumps, in which case the mouth should be cleared of blood by a sponge from time to time, until the operation is completed. It is better to do this than allow the patient to recover before the completion of the operation or be allowed to swallow the blood, which frequently produces nausea and sickness.

Some dental practitioners have advised to place a piece of cork, or wedge of wood, between the teeth before commencing the inhalation, to act as a gag and keep the mouth open ready for the operator. I do not recommend this practice, because

*I recently had a patient, a gentleman, who inhaled six drachms of chloroform before perfect insensibility was produced.

the patient almost invariably exhibits some degree of nervousness when presenting himself for an operation, and the less he is reminded of his position the better, by making it as simple an affair as possible. Should he experience any difficulty in opening the mouth, we advise him to employ a wedge made of some soft wood, and gently insert it between the teeth, at one side of the mouth, in preference to running the risk of injuring the front incisors, which is very likely to occur, if they be forced between them. In my practice, I seldom have occasion to employ any mechanical means of opening the mouth. As regards the dangerous effects that may occur from the inhalation of anæsthetic agents, no doubt, we believe, exists, that there are particular diseases and idiosyncrasies of constitution in which it would not be advisable to administer it, but these we believe are very rare. Medical authorities, generally, concur in opinion that it should not be exhibited in patients suffering from diseases of the heart, pulmonary affections, determination of blood to the head, or who have a very slow and languid circulation, except for some severe and protracted surgical operation. We advise the young practitioner before he ventures to administer either of these agents, to make himself perfectly acquainted with the modus operandi, to attend repeatedly the public operations at our hospitals, and closely observe its exhibition and the various degrees of narcotization before and after an operation, watch the period at which the patient becomes sufficiently insensible for the commencement of the operation, and the state of the pulse at the various stages of its exhibition. He ought to become perfectly familiar with these particulars, and test his knowledge of the subject, by practicing upon some of the lower animals before he ventures to administer it to a human subject. By following this course he will soon obtain confidence in the exhibition of the agents in his operations under its influence in private practice.

We should advise every young practitioner, before administering anæsthetic agents, to have the opinion of the patient's medical adviser, and if possible his assistance. It is impossible, in a protracted dental operation, for the operator to attend to the pulse, administer the agent, observe the index, perform the operation and combat all the difficulties which often occur in the extraction of teeth.

*Purity of Ether and Chloroform.*—When ether and chloroform were first introduced, I experienced considerable difficulty in procuring a perfectly pure article from the chemists. It is of the utmost importance that great attention should be paid to the purity and uniform strength of these agents, for if our materials be impure, we shall unquestionably have different results. Ether should be of the specific gravity of about 725, and perfectly free from any sulphuric acid. The specific gravity of chloroform should be 1.48, it should possess an agreeable and fruity odor, and be perfectly free from chlorine and alcohol, either of which cause considerable irritation during inspiration, and produce nausea, head-ache, &c. To detect some of these impurities in chloroform, chromic acid has been recommended by Dr. Cattell for the detection of alcohol. If there be but one drop of the alcohol in a large amount of chloroform, the reaction of chromic acid on the alcohol produces the beautiful green oxyd of chrome. This test is positively indicative of the presence of alcohol, and consequently, of the impurity of chloroform. Chlorine might be readily detected by dropping in the suspected fluid a crystal of nitrate of silver—if free chlorine be present, the fluid will assume a turbid appearance and precipitate of chloride of silver will soon fall, which becomes black by exposure to light.

*Anæsthesia, produced by Cold.*—Since the introduction of ether and chloroform, Dr. Arnott, of Brighton, has directed the attention of the profession by the publication of several papers upon anæsthesia produced by the local application of ice and salt; since which, there has been several successful operations reported in the "medical journals." In consequence of these reports, many dental practitioners attempted the extraction of teeth, by the local application of ice and salt enclosed in some thin kind of membrane. From the favorable reports in surgical operations, I was induced to make trial of the new anæsthesia at the "Royal Free Hospital," as well as in private practice; in fourteen cases in which I applied the cold, eight of the patients were suffering intense pain from exposed pulps; the remaining six cases in which it was applied, were to necrosed stumps of teeth. For the purpose of these trials, I used the inner coat of the stomach of a fowl, and also the skin of a common sausage. Upon the introduction of the cold in the eight first cases, the intense pain produced was indescribable, so much so, that in five out of the number, I was obliged to desist, as the pain was unbearable, and I was compelled to extract the teeth at once. The remaining three patients were determined to submit, and ultimately I succeeded in the removal of the teeth without pain. The time occupied varied from five to ten minutes, the application of fresh ice, which was ready at hand, being necessary every five or six minutes. In the application of the cold to the stump cases, little or no pain was experienced, excepting when the cold came in contact with any of the teeth, the whole of these I might fairly state were satisfactory. In the application I experienced considerable annoyance, by the rupture of the membrane containing the salt and ice, a great portion of which was swallowed by the patient, producing results anything but agreeable, considering that salt and water forms an active emetic.

As far as the mere extraction of the teeth was concerned, the application of the cold was so satisfactory in the three cases referred to, of course leaving out of view the intense suffering produced by the application, before I could operate, but in two cases out of the three, the hemorrhage produced from the reaction was alarming, and in one instance, I questioned the safety of the patient for upwards of thirty-six hours; the bleeding that followed the removal of the stumps, was not more than is generally observed in ordinary cases. From the preceding observations, it is clear, that for general application, in dental surgery, the new anæsthesia is not at present complete—but to obviate or lessen some of those difficulties, I am having constructed a contrivance by means of which the temperature of the fluid first introduced shall be the same as the cavity of the mouth—this I propose doing by suspending a metallic vessel as a reservoir for containing the freezing mixture. At the bottom of this vessel is secured a vulcanized India rubber tube, of the required length and diameter, at the end of which is a stopcock. The part of the apparatus proposed for the mouth, will consist of two wooden tubes, one end of which is of sufficient size for receiving the end of the stopcock, attached to the reservoir tube—to the other tube or pipe (which is separate) is attached a common bladder with stopcock, and a piece of vulcanized tube—the object of the bladder is to act as another reservoir. When the whole of the apparatus is complete, to make the connexion, we shall secure a piece of thin membrane at the end of the tubes—which will, when filled, form a bag by filling the tube which is to be attached to the reservoir, with warm water, and fixing it on the stopcock; it will be seen we have now only warm water to apply to the mouth, by turning the stopcock, a supply of cold can be gradually introduced, which will displace the warm fluid, by forcing it into the bladder, and continued until the parts are sufficiently frozen to admit of operating. My object in furnishing these details at the present time to the profession is, with the view of enlisting the co-operation of the members of the dental art in America in the investigation of this new anæsthetic agent. It has been rumored in England, that some dentist has applied to the patent office to obtain letters patent for some similar application. Upon inquiries being made at the office, it was found that a specification had been sent in, but of course as all documents of this character are for a time only "signed and sealed," no opportunity at present exists to enable the profession to judge in what possible way the would-be patentee can frame his future application for the patent. We think this "hint" to the scientific dentists of America will be sufficient to prevent a similar piece of "doing trade" being carried into effect in their country; this mania for patent rights by professional men, I hope to see discontinued, both in England and America—or otherwise we shall not be surprised some fine morning to find onesself paying a "trading tax" for an *improved* method for boiling eggs—and another for eating them.

